# Supplementary figures and images for: Micro-costing for national-scale azithromycin mass drug administration to improve child survival in Niger
Source: PLOS Glob Public Health. 2026 Jun 26;6(6):e0006039. doi: 10.1371/journal.pgph.0006039 (PMC13309011; doi:10.1371/journal.pgph.0006039)

# Supplemental Figure 1: Stacked Bar Graph of Percentage Cost Breakdown

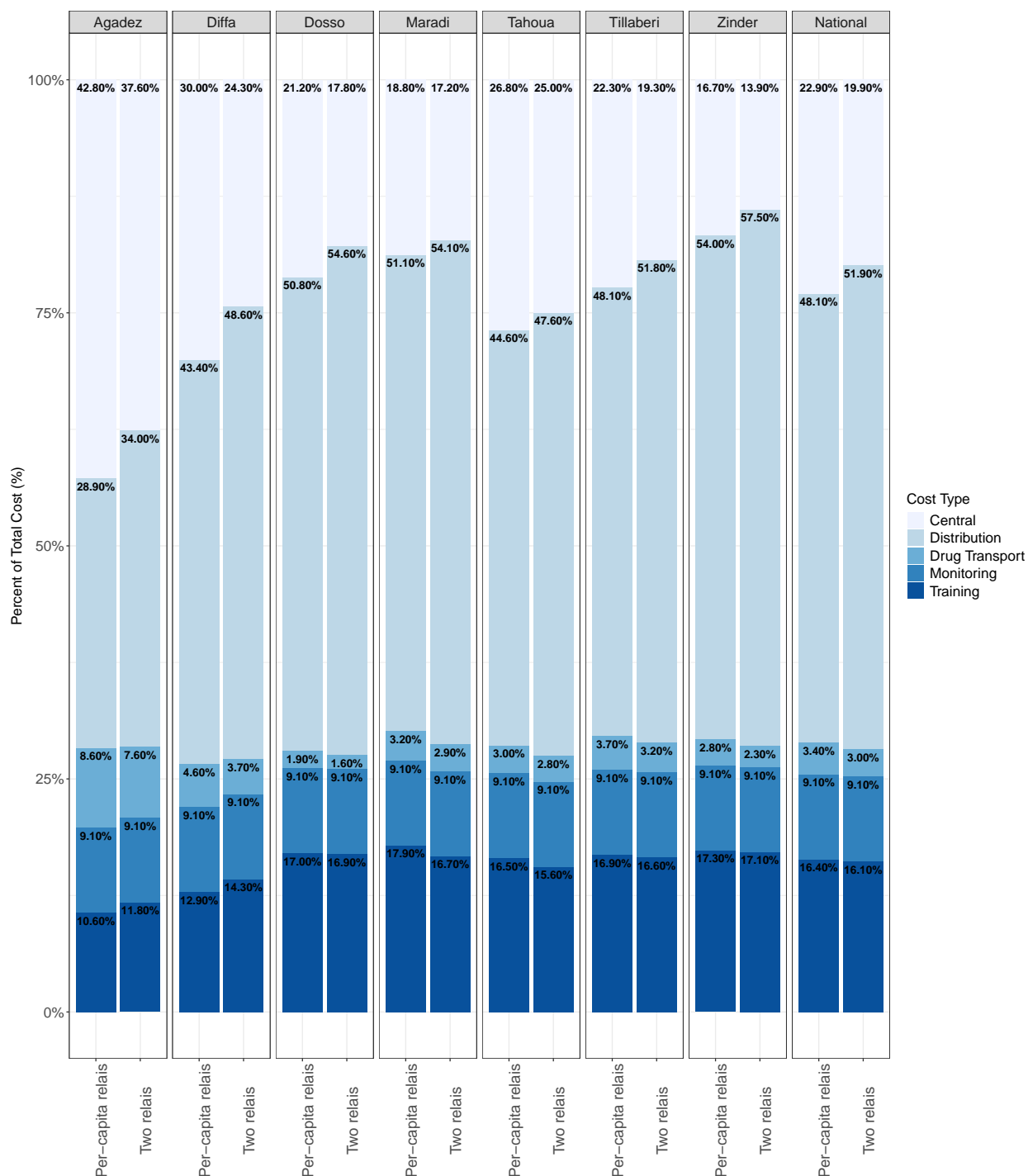

Supplement: S1 Fig — (PDF) [file pgph.0006039.s001.pdf]

Supplemental Figure 2. Price per Percent of CHW Capacity

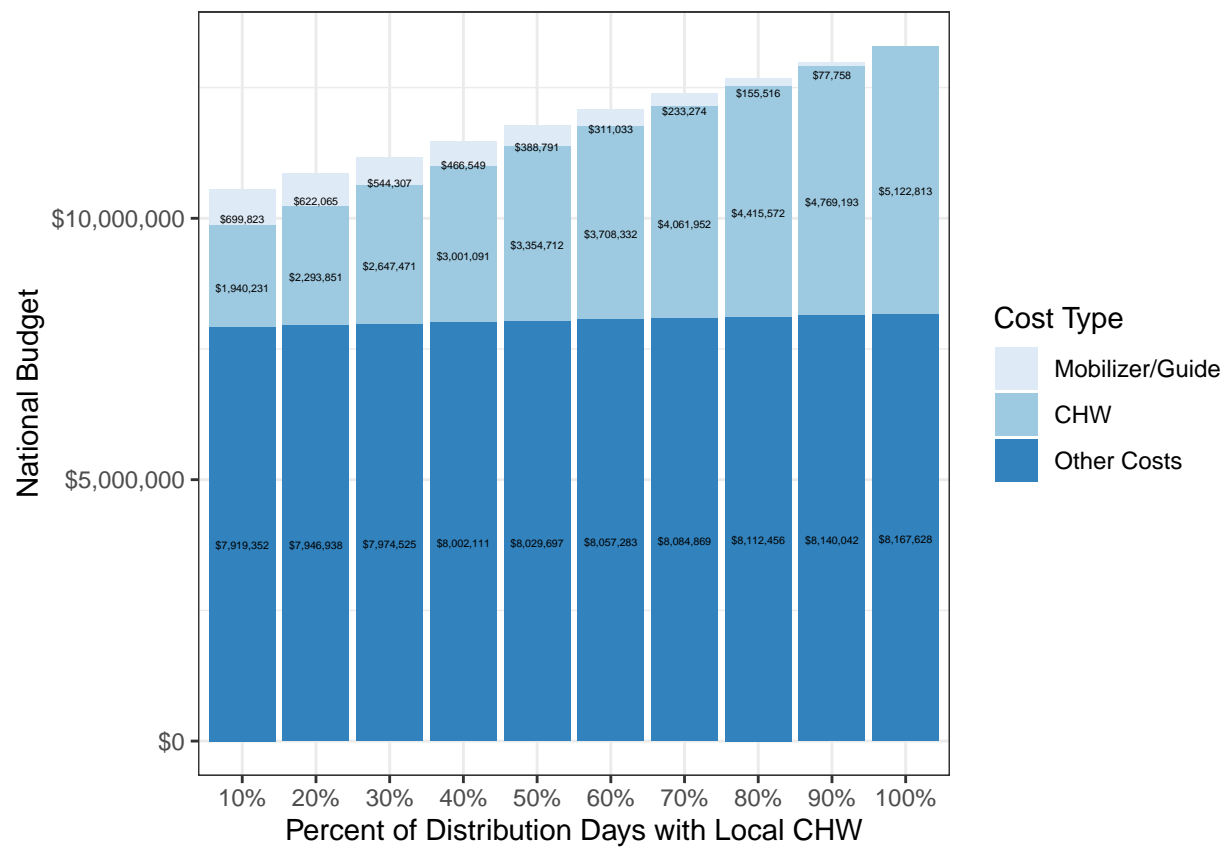

Supplement: S2 Fig — (PDF) [file pgph.0006039.s002.pdf]
