# Supplementary material for: Micro-costing for national-scale azithromycin mass drug administration to improve child survival in Niger
Source: PLOS Glob Public Health. 2026 Jun 26;6(6):e0006039. doi: 10.1371/journal.pgph.0006039 (PMC13309011; doi:10.1371/journal.pgph.0006039)
Supplement: S1 Table — (PDF) [file pgph.0006039.s003.pdf]

Supplemental Table 1. Distribution costs by item

Item	Dosso	Tahoua	Maradi	Zinder	Tillaberi	Agadez	Diffa	National
Backpacks	$30,828 ($29,995, $35,828)	$31,205 ($30,362, $36,266)	$40,657 ($39,558, $47,250)	$53,056 ($51,622, $61,660)	$39,265 ($38,204, $45,633)	$6,503.93 ($6,328.15, $7,558.63)	$13,868 ($13,493, $16,117)	$215,384 ($209,562, $250,311)
Mineral Water	$3,132 ($2,262, $3,480)	$5,086 ($3,673, $5,651)	$5,183 ($3,743, $5,759)	$5,400 ($3,900, $6,000)	$4,154 ($3,000, $4,616)	$714.66 ($516.14, $794.07)	$871 ($629, $967)	$24,540 ($17,724, $27,267)
Hand Sanitizer	$41,737 ($41,737, $41,737)	$42,247 ($42,247, $42,247)	$55,042 ($55,042, $55,042)	$71,829 ($71,829, $71,829)	$53,158 ($53,158, $53,158)	$8,805.23 ($8,805.23, $8,805.23)	$18,775 ($18,775, $18,775)	$291,593 ($291,593, $291,593)
Dosing poles	$54,021 ($52,560, $62,781)	$54,681 ($53,203, $63,548)	$71,243 ($69,317, $82,796)	$92,969 ($90,457, $108,046)	$68,804 ($66,945, $79,962)	$11,396.80 ($11,088.78, $13,244.93)	$24,301 ($23,644, $28,241)	$377,415 ($367,215, $438,618)
Syringes	$16,440 ($11,873, $18,267)	$26,695 ($19,279, $29,661)	$27,202 ($19,646, $30,224)	$28,339 ($20,467, $31,488)	$21,802 ($15,746, $24,225)	$3,750.77 ($2,708.89, $4,167.52)	$4,569 ($3,300, $5,077)	$128,798 ($93,021, $143,109)
Chief per diem	$107,145 ($107,145, $107,145)	$89,959 ($89,959, $89,959)	$118,058 ($118,058, $118,058)	$183,273 ($183,273, $183,273)	$130,931 ($130,931, $130,931)	$26,486.85 ($26,486.85, $26,486.85)	$57,330 ($57,330, $57,330)	$713,185 ($713,185, $713,185)
Mobiliser/Guide per diem	$28,937 ($15,582, $37,841)	$29,291 ($15,772, $31,544)	$38,163 ($20,549, $41,098)	$49,801 ($26,816, $53,632)	$36,857 ($19,846, $39,692)	$6,104.96 ($3,287.28, $6,574.57)	$13,017 ($7,009, $14,019)	$202,171 ($108,861, $224,401)
Relais per diem	$227,096 ($227,096, $227,096)	$229,872 ($229,872, $229,872)	$299,496 ($299,496, $299,496)	$390,833 ($390,833, $390,833)	$289,245 ($289,245, $289,245)	$47,910.84 ($47,910.84, $47,910.84)	$102,158 ($102,158, $102,158)	$1,586,611 ($1,586,611, $1,586,611)
Relais transportation	$61,657 ($59,991, $71,655)	$62,411 ($60,724, $72,531)	$81,314 ($79,116, $94,500)	$106,112 ($103,244, $123,319)	$78,530 ($76,408, $91,265)	$13,007.87 ($12,656.30, $15,117.25)	$27,736 ($26,986, $32,234)	$430,767 ($419,125, $500,621)
Case agent	$22,324 ($22,324, $22,324)	$22,385 ($22,385, $22,385)	$25,496 ($25,496, $25,496)	$26,716 ($26,716, $26,716)	$33,914 ($33,914, $33,914)	$11,467.16 ($11,467.16, $11,467.16)	$22,324 ($22,324, $22,324)	$164,627 ($164,627, $164,627)
CSI Chief per diem	$7,973 ($7,973, $7,973)	$11,589 ($11,589, $11,589)	$9,106 ($9,106, $9,106)	$9,541 ($9,541, $9,541)	$12,112 ($12,112, $12,112)	$4,095.42 ($4,095.42, $4,095.42)	$3,137 ($3,137, $3,137)	$57,554 ($57,554, $57,554)

CSI Chief communication	$1,993 ($1,993, $1,993)	$2,897 ($2,897, $2,897)	$2,276 ($2,276, $2,276)	$2,385 ($2,385, $2,385)	$3,028 ($3,028, $3,028)	$1,023.85 ($1,023.85, $1,023.85)	$784 ($784, $784)	$14,388 ($14,388, $14,388)
Per diem National authorities	$34 ($34, $34)	$34 ($34, $34)	$34 ($34, $34)	$34 ($34, $34)	$34 ($34, $34)	$34.23 ($34.23, $34.23)	$34 ($34, $34)	$240 ($240, $240)
Per diem Regional authorities	$240 ($240, $240)	$240 ($240, $240)	$240 ($240, $240)	$240 ($240, $240)	$240 ($240, $240)	$239.63 ($239.63, $239.63)	$240 ($240, $240)	$1,677 ($1,677, $1,677)
Per diems District authorities	$1,150 ($1,150, $1,150)	$1,869 ($1,869, $1,869)	$1,294 ($1,294, $1,294)	$1,582 ($1,582, $1,582)	$1,869 ($1,869, $1,869)	$1,006.43 ($1,006.43, $1,006.43)	$863 ($863, $863)	$9,633 ($9,633, $9,633)
Per diems CSI Leaders	$8,770 ($8,770, $8,770)	$12,748 ($12,748, $12,748)	$10,016 ($10,016, $10,016)	$10,496 ($10,496, $10,496)	$13,323 ($13,323, $13,323)	$4,504.96 ($4,504.96, $4,504.96)	$3,451 ($3,451, $3,451)	$63,309 ($63,309, $63,309)
Per diems Local authorities	$235,743 ($235,743, $235,743)	$197,931 ($197,931, $197,931)	$259,754 ($259,754, $259,754)	$403,242 ($403,242, $403,242)	$288,078 ($288,078, $288,078)	$58,276.90 ($58,276.90, $58,276.90)	$126,139 ($126,139, $126,139)	$1,569,163 ($1,569,163, $1,569,163)
Radio communication	$4,793 ($4,793, $4,793)	$7,788 ($7,788, $7,788)	$5,392 ($5,392, $5,392)	$6,590 ($6,590, $6,590)	$7,788 ($7,788, $7,788)	$4,193.44 ($4,193.44, $4,193.44)	$3,594 ($3,594, $3,594)	$40,137 ($40,137, $40,137)
Support for communication	$4,793 ($4,793, $4,793)	$7,788 ($7,788, $7,788)	$5,392 ($5,392, $5,392)	$6,590 ($6,590, $6,590)	$7,788 ($7,788, $7,788)	$4,193.44 ($4,193.44, $4,193.44)	$3,594 ($3,594, $3,594)	$40,137 ($40,137, $40,137)
Refreshments	$4,793 ($4,793, $4,793)	$7,788 ($7,788, $7,788)	$5,392 ($5,392, $5,392)	$6,590 ($6,590, $6,590)	$7,788 ($7,788, $7,788)	$4,193.44 ($4,193.44, $4,193.44)	$3,594 ($3,594, $3,594)	$40,137 ($40,137, $40,137)
Mapping	$6,735 ($5,509, $7,959)	$6,735 ($5,509, $7,959)	$6,735 ($5,509, $7,959)	$6,735 ($5,509, $7,959)	$6,735 ($5,509, $7,959)	$6,734.67 ($5,509.24, $7,958.99)	$6,735 ($5,509, $7,959)	$47,143 ($38,565, $55,713)
