# Supplementary material for: Micro-costing for national-scale azithromycin mass drug administration to improve child survival in Niger
Source: PLOS Glob Public Health. 2026 Jun 26;6(6):e0006039. doi: 10.1371/journal.pgph.0006039 (PMC13309011; doi:10.1371/journal.pgph.0006039)
Supplement: S2 Table — (PDF) [file pgph.0006039.s004.pdf]

Supplemental Table 2. Training costs by item

Item	Dosso	Tahoua	Maradi	Zinder	Tillaberi	Agadez	Diffa	National
Supervisor	$697.02 ($697.02, $697.02)	$1,132.66 ($1,132.66, $1,132.66)	$784 ($784, $784)	$958 ($958, $958)	$1,133 ($1,133, $1,133)	$609.89 ($609.89, $609.89)	$522.77 ($522.77, $522.77)	$5,838 ($5,838, $5,838)
Room rental	$1,742.75 ($1,742.75, $1,742.75)	$2,831.96 ($2,831.96, $2,831.96)	$1,961 ($1,961, $1,961)	$2,396 ($2,396, $2,396)	$2,832 ($2,832, $2,832)	$1,524.90 ($1,524.90, $1,524.90)	$1,307.06 ($1,307.06, $1,307.06)	$14,596 ($14,596, $14,596)
District chief communication	$1,394.04 ($1,394.04, $1,394.04)	$2,265.32 ($2,265.32, $2,265.32)	$1,568 ($1,568, $1,568)	$1,917 ($1,917, $1,917)	$2,265 ($2,265, $2,265)	$1,219.79 ($1,219.79, $1,219.79)	$1,045.53 ($1,045.53, $1,045.53)	$11,675 ($11,675, $11,675)
District chief meals and coffee	$174.26 ($174.26, $174.26)	$283.17 ($283.17, $283.17)	$196 ($196, $196)	$240 ($240, $240)	$283 ($283, $283)	$152.47 ($152.47, $152.47)	$130.69 ($130.69, $130.69)	$1,459 ($1,459, $1,459)
District chief per diem	$1,045.73 ($1,045.73, $1,045.73)	$1,699.30 ($1,699.30, $1,699.30)	$1,176 ($1,176, $1,176)	$1,438 ($1,438, $1,438)	$1,699 ($1,699, $1,699)	$915.01 ($915.01, $915.01)	$784.29 ($784.29, $784.29)	$8,758 ($8,758, $8,758)
District communicator communication	$348.51 ($348.51, $348.51)	$566.33 ($566.33, $566.33)	$392 ($392, $392)	$479 ($479, $479)	$566 ($566, $566)	$304.95 ($304.95, $304.95)	$261.38 ($261.38, $261.38)	$2,919 ($2,919, $2,919)
CSI Chief per diem (step 1)	$15,944.39 ($15,944.39, $15,944.39)	$23,175.99 ($23,175.99, $23,175.99)	$18,210 ($18,210, $18,210)	$19,081 ($19,081, $19,081)	$24,222 ($24,222, $24,222)	$8,190.01 ($8,190.01, $8,190.01)	$6,273.20 ($6,273.20, $6,273.20)	$115,096 ($115,096, $115,096)
CSI Chief per diem (step 2)	$7,972.19 ($7,972.19, $7,972.19)	$11,588.00 ($11,588.00, $11,588.00)	$9,105 ($9,105, $9,105)	$9,540 ($9,540, $9,540)	$12,111 ($12,111, $12,111)	$4,095.01 ($4,095.01, $4,095.01)	$3,136.60 ($3,136.60, $3,136.60)	$57,548 ($57,548, $57,548)
CSI Chief communication	$1,995.24 ($1,995.24, $1,995.24)	$2,900.19 ($2,900.19, $2,900.19)	$2,279 ($2,279, $2,279)	$2,388 ($2,388, $2,388)	$3,031 ($3,031, $3,031)	$1,024.88 ($1,024.88, $1,024.88)	$785.01 ($785.01, $785.01)	$14,403 ($14,403, $14,403)
CSI chief meals and coffee (step 1)	$3,986.10 ($3,986.10, $3,986.10)	$5,794.00 ($5,794.00, $5,794.00)	$4,552 ($4,552, $4,552)	$4,770 ($4,770, $4,770)	$6,055 ($6,055, $6,055)	$2,047.50 ($2,047.50, $2,047.50)	$1,568.30 ($1,568.30, $1,568.30)	$28,774 ($28,774, $28,774)
CSI chief meals and coffee (step 2)	$1,995.24 ($1,995.24, $1,995.24)	$2,900.19 ($2,900.19, $2,900.19)	$2,279 ($2,279, $2,279)	$2,388 ($2,388, $2,388)	$3,031 ($3,031, $3,031)	$1,024.88 ($1,024.88, $1,024.88)	$785.01 ($785.01, $785.01)	$14,403 ($14,403, $14,403)

CSI chief transportation	$6,380.39 ($6,380.39, $6,380.39)	$9,274.22 ($9,274.22, $9,274.22)	$7,287 ($7,287, $7,287)	$7,636 ($7,636, $7,636)	$9,693 ($9,693, $9,693)	$3,277.36 ($3,277.36, $3,277.36)	$2,510.32 ($2,510.32, $2,510.32)	$46,057 ($46,057, $46,057)
Case agent per diem	$11,162.19 ($11,162.19, $11,162.19)	$11,192.68 ($11,192.68, $11,192.68)	$12,748 ($12,748, $12,748)	$13,358 ($13,358, $13,358)	$16,957 ($16,957, $16,957)	$5,733.58 ($5,733.58, $5,733.58)	$4,391.68 ($4,391.68, $4,391.68)	$75,543 ($75,543, $75,543)
Case agent meals and coffee	$3,990.48 ($3,990.48, $3,990.48)	$4,001.38 ($4,001.38, $4,001.38)	$4,557 ($4,557, $4,557)	$4,775 ($4,775, $4,775)	$6,062 ($6,062, $6,062)	$2,049.76 ($2,049.76, $2,049.76)	$1,570.03 ($1,570.03, $1,570.03)	$27,007 ($27,007, $27,007)
Case agent transportation	$4,779.81 ($4,779.81, $4,779.81)	$4,792.87 ($4,792.87, $4,792.87)	$5,459 ($5,459, $5,459)	$5,720 ($5,720, $5,720)	$7,261 ($7,261, $7,261)	$2,455.20 ($2,455.20, $2,455.20)	$1,880.58 ($1,880.58, $1,880.58)	$32,349 ($32,349, $32,349)
Relais meals and coffee (step 2)	$51,475.03 ($50,083.82, $59,822.34)	$52,104.35 ($50,696.13, $60,553.71)	$67,886 ($66,051, $78,894)	$88,589 ($86,194, $102,954)	$65,562 ($63,790, $76,194)	$10,859.78 ($10,566.27, $12,620.83)	$23,155.70 ($22,529.87, $26,910.68)	$359,631 ($349,912, $417,950)
Relais notebooks and pens	$30,885.02 ($30,050.29, $35,893.40)	$31,262.61 ($30,417.68, $36,332.22)	$40,731 ($39,631, $47,337)	$53,153 ($51,717, $61,773)	$39,337 ($38,274, $45,716)	$6,515.87 ($6,339.76, $7,572.50)	$13,893.42 ($13,517.92, $16,146.41)	$215,779 ($209,947, $250,770)
Relais per diem	$84,025.51 ($81,754.55, $97,651.27)	$85,052.78 ($82,754.05, $98,845.12)	$110,814 ($107,819, $128,783)	$144,608 ($140,700, $168,058)	$107,021 ($104,128, $124,375)	$17,727.01 ($17,247.90, $20,601.66)	$37,798.31 ($36,776.73, $43,927.76)	$587,046 ($571,180, $682,243)
Relais transportation	$61,656.91 ($59,990.51, $71,655.33)	$62,410.71 ($60,723.93, $72,531.36)	$81,314 ($79,116, $94,500)	$106,112 ($103,244, $123,319)	$78,530 ($76,408, $91,265)	$13,007.87 ($12,656.30, $15,117.25)	$27,735.94 ($26,986.32, $32,233.67)	$430,767 ($419,125, $500,621)
