# Supplementary material for: Micro-costing for national-scale azithromycin mass drug administration to improve child survival in Niger
Source: PLOS Glob Public Health. 2026 Jun 26;6(6):e0006039. doi: 10.1371/journal.pgph.0006039 (PMC13309011; doi:10.1371/journal.pgph.0006039)
Supplement: S3 Table — (PDF) [file pgph.0006039.s005.pdf]

Supplemental Table 3. Drug transport and cold chain storage costs by item

Item	Dosso	Tahoua	Maradi	Zinder	Tillaberi	Agadez	Diffa	National
Cost to bring treatment to CSIs	$5,199.41 ($5,199.41, $5,199.41)	$10,365.90 ($10,365.90, $10,365.90)	$13,260.50 ($13,260.50, $13,260.50)	$16,336.47 ($16,336.47, $16,336.47)	$22,027.76 ($22,027.76, $22,027.76)	$22,344.50 ($22,344.50, $22,344.50)	$14,732.25 ($14,732.25, $14,732.25)	$104,267 ($104,267, $104,267)
Cost to retrieve treatment and do inventory	$9,358.92 ($9,358.92, $9,358.92)	$18,658.61 ($18,658.61, $18,658.61)	$23,868.91 ($23,868.91, $23,868.91)	$29,405.65 ($29,405.65, $29,405.65)	$39,649.96 ($39,649.96, $39,649.96)	$40,220.10 ($40,220.10, $40,220.10)	$26,518.05 ($26,518.05, $26,518.05)	$187,680 ($187,680, $187,680)
Cost for quality control of treatment	$158.09 ($158.09, $158.09)	$158.09 ($158.09, $158.09)	$158.09 ($158.09, $158.09)	$158.09 ($158.09, $158.09)	$158.09 ($158.09, $158.09)	$158.09 ($158.09, $158.09)	$158.09 ($158.09, $158.09)	$1,107 ($1,107, $1,107)
Cost for storage	$17,338.72 ($12,588.38, $19,238.85)	$28,026.97 ($20,426.44, $31,352.20)	$28,739.52 ($20,663.95, $31,827.24)	$29,927.10 ($21,614.02, $33,252.34)	$23,039.12 ($16,626.17, $25,651.80)	$4,037.78 ($2,850.20, $4,512.82)	$4,987.85 ($3,562.75, $5,462.88)	$136,097 ($98,332, $151,298)
