# Supplementary material for: Micro-costing for national-scale azithromycin mass drug administration to improve child survival in Niger
Source: PLOS Glob Public Health. 2026 Jun 26;6(6):e0006039. doi: 10.1371/journal.pgph.0006039 (PMC13309011; doi:10.1371/journal.pgph.0006039)
Supplement: S4 Table — (PDF) [file pgph.0006039.s006.pdf]

Supplemental Table 4. Central costs by item

Item	Dosso	Tahoua	Maradi	Zinder	Tillaberi	Agadez	Diffa	National
Chaffeur - director and subdirector	$1,867.37 ($1,867.37, $1,867.37)	$1,867 ($1,867, $1,867)	$1,867.37 ($1,867.37, $1,867.37)	$1,867 ($1,867, $1,867)	$1,867 ($1,867, $1,867)	$1,867.37 ($1,867.37, $1,867.37)	$1,867.37 ($1,867.37, $1,867.37)	$13,072 ($13,072, $13,072)
Cost agent	$1,680.46 ($1,680.46, $1,680.46)	$1,680 ($1,680, $1,680)	$1,680.46 ($1,680.46, $1,680.46)	$1,680 ($1,680, $1,680)	$1,680 ($1,680, $1,680)	$1,680.46 ($1,680.46, $1,680.46)	$1,680.46 ($1,680.46, $1,680.46)	$11,763 ($11,763, $11,763)
National office internet	$224.05 ($224.05, $224.05)	$224 ($224, $224)	$224.05 ($224.05, $224.05)	$224 ($224, $224)	$224 ($224, $224)	$224.05 ($224.05, $224.05)	$224.05 ($224.05, $224.05)	$1,568 ($1,568, $1,568)
National office space rent	$2,987.62 ($2,987.62, $2,987.62)	$2,988 ($2,988, $2,988)	$2,987.62 ($2,987.62, $2,987.62)	$2,988 ($2,988, $2,988)	$2,988 ($2,988, $2,988)	$2,987.62 ($2,987.62, $2,987.62)	$2,987.62 ($2,987.62, $2,987.62)	$20,913 ($20,913, $20,913)
National office water	$1,374.25 ($1,374.25, $1,374.25)	$1,374 ($1,374, $1,374)	$1,374.25 ($1,374.25, $1,374.25)	$1,374 ($1,374, $1,374)	$1,374 ($1,374, $1,374)	$1,374.25 ($1,374.25, $1,374.25)	$1,374.25 ($1,374.25, $1,374.25)	$9,620 ($9,620, $9,620)
Vehicle fuel	$1,997.45 ($1,997.45, $1,997.45)	$1,997 ($1,997, $1,997)	$1,997.45 ($1,997.45, $1,997.45)	$1,997 ($1,997, $1,997)	$1,997 ($1,997, $1,997)	$1,997.45 ($1,997.45, $1,997.45)	$1,997.45 ($1,997.45, $1,997.45)	$13,982 ($13,982, $13,982)
Vehicle insurance	$622.34 ($622.34, $622.34)	$622 ($622, $622)	$622.34 ($622.34, $622.34)	$622 ($622, $622)	$622 ($622, $622)	$622.34 ($622.34, $622.34)	$622.34 ($622.34, $622.34)	$4,356 ($4,356, $4,356)
Vehicle maintenance	$404.62 ($404.62, $404.62)	$405 ($405, $405)	$404.62 ($404.62, $404.62)	$405 ($405, $405)	$405 ($405, $405)	$404.62 ($404.62, $404.62)	$404.62 ($404.62, $404.62)	$2,832 ($2,832, $2,832)
Program director	$16,653.28 ($16,653.28, $16,653.28)	$16,653 ($16,653, $16,653)	$16,653.28 ($16,653.28, $16,653.28)	$16,653 ($16,653, $16,653)	$16,653 ($16,653, $16,653)	$16,653.28 ($16,653.28, $16,653.28)	$16,653.28 ($16,653.28, $16,653.28)	$116,573 ($116,573, $116,573)
Program sub-director	$13,083.55 ($13,083.55, $13,083.55)	$13,084 ($13,084, $13,084)	$13,083.55 ($13,083.55, $13,083.55)	$13,084 ($13,084, $13,084)	$13,084 ($13,084, $13,084)	$13,083.55 ($13,083.55, $13,083.55)	$13,083.55 ($13,083.55, $13,083.55)	$91,585 ($91,585, $91,585)
Vehicle - director and subdirector	$2,881.73 ($2,881.73, $2,881.73)	$2,882 ($2,882, $2,882)	$2,881.73 ($2,881.73, $2,881.73)	$2,882 ($2,882, $2,882)	$2,882 ($2,882, $2,882)	$2,881.73 ($2,881.73, $2,881.73)	$2,881.73 ($2,881.73, $2,881.73)	$20,172 ($20,172, $20,172)

Data manager	$11,763.21 ($11,763.21, $11,763.21)	$11,763 ($11,763, $11,763)	$11,763.21 ($11,763.21, $11,763.21)	$11,763 ($11,763, $11,763)	$11,763 ($11,763, $11,763)	$11,763.21 ($11,763.21, $11,763.21)	$11,763.21 ($11,763.21, $11,763.21)	$82,342 ($82,342, $82,342)
Logisitics vehicle driver	$13,071.57 ($13,071.57, $13,071.57)	$13,072 ($13,072, $13,072)	$13,071.57 ($13,071.57, $13,071.57)	$13,072 ($13,072, $13,072)	$13,072 ($13,072, $13,072)	$13,071.57 ($13,071.57, $13,071.57)	$13,071.57 ($13,071.57, $13,071.57)	$91,501 ($91,501, $91,501)
Vehicle fuel	$13,982.14 ($13,982.14, $13,982.14)	$13,982 ($13,982, $13,982)	$13,982.14 ($13,982.14, $13,982.14)	$13,982 ($13,982, $13,982)	$13,982 ($13,982, $13,982)	$13,982.14 ($13,982.14, $13,982.14)	$13,982.14 ($13,982.14, $13,982.14)	$97,875 ($97,875, $97,875)
Vehicle insurance	$4,356.39 ($4,356.39, $4,356.39)	$4,356 ($4,356, $4,356)	$4,356.39 ($4,356.39, $4,356.39)	$4,356 ($4,356, $4,356)	$4,356 ($4,356, $4,356)	$4,356.39 ($4,356.39, $4,356.39)	$4,356.39 ($4,356.39, $4,356.39)	$30,495 ($30,495, $30,495)
Vehicle maintenance	$2,832.37 ($2,832.37, $2,832.37)	$2,832 ($2,832, $2,832)	$2,832.37 ($2,832.37, $2,832.37)	$2,832 ($2,832, $2,832)	$2,832 ($2,832, $2,832)	$2,832.37 ($2,832.37, $2,832.37)	$2,832.37 ($2,832.37, $2,832.37)	$19,827 ($19,827, $19,827)
Regional coordinator	$11,763.21 ($11,763.21, $11,763.21)	$11,763 ($11,763, $11,763)	$11,763.21 ($11,763.21, $11,763.21)	$11,763 ($11,763, $11,763)	$11,763 ($11,763, $11,763)	$11,763.21 ($11,763.21, $11,763.21)	$11,763.21 ($11,763.21, $11,763.21)	$82,342 ($82,342, $82,342)
Vehicle logistics agents	$20,172.10 ($20,172.10, $20,172.10)	$20,172 ($20,172, $20,172)	$20,172.10 ($20,172.10, $20,172.10)	$20,172 ($20,172, $20,172)	$20,172 ($20,172, $20,172)	$20,172.10 ($20,172.10, $20,172.10)	$20,172.10 ($20,172.10, $20,172.10)	$141,205 ($141,205, $141,205)
Supervisor	$75,290.30 ($75,290.30, $75,290.30)	$122,347 ($122,347, $122,347)	$84,701.59 ($84,701.59, $84,701.59)	$103,524 ($103,524, $103,524)	$122,347 ($122,347, $122,347)	$65,879.01 ($65,879.01, $65,879.01)	$56,467.73 ($56,467.73, $56,467.73)	$630,556 ($630,556, $630,556)
Vehicle insurance	$17,425.56 ($17,425.56, $17,425.56)	$28,317 ($28,317, $28,317)	$19,603.75 ($19,603.75, $19,603.75)	$23,960 ($23,960, $23,960)	$28,317 ($28,317, $28,317)	$15,247.36 ($15,247.36, $15,247.36)	$13,069.17 ($13,069.17, $13,069.17)	$145,939 ($145,939, $145,939)
Vehicle maintenance	$11,329.49 ($11,329.49, $11,329.49)	$18,410 ($18,410, $18,410)	$12,745.67 ($12,745.67, $12,745.67)	$15,578 ($15,578, $15,578)	$18,410 ($18,410, $18,410)	$9,913.30 ($9,913.30, $9,913.30)	$8,497.12 ($8,497.12, $8,497.12)	$94,884 ($94,884, $94,884)
Vehicle director	$80,688.41 ($80,688.41, $80,688.41)	$131,119 ($131,119, $131,119)	$90,774.46 ($90,774.46, $90,774.46)	$110,947 ($110,947, $110,947)	$131,119 ($131,119, $131,119)	$70,602.36 ($70,602.36, $70,602.36)	$60,516.31 ($60,516.31, $60,516.31)	$675,765 ($675,765, $675,765)
Vehicle Fuel	$55,928.57 ($55,928.57, $55,928.57)	$90,884 ($90,884, $90,884)	$62,919.64 ($62,919.64, $62,919.64)	$76,902 ($76,902, $76,902)	$90,884 ($90,884, $90,884)	$48,937.50 ($48,937.50, $48,937.50)	$41,946.43 ($41,946.43, $41,946.43)	$468,402 ($468,402, $468,402)
