# Supplementary material for: Micro-costing for national-scale azithromycin mass drug administration to improve child survival in Niger
Source: PLOS Glob Public Health. 2026 Jun 26;6(6):e0006039. doi: 10.1371/journal.pgph.0006039 (PMC13309011; doi:10.1371/journal.pgph.0006039)
Supplement: S5 Table — (PDF) [file pgph.0006039.s007.pdf]

## Supplemental Table 5. Monitoring costs by item

| Item | Dosso | Tahoua | Maradi | Zinder | Tillaberi | Agadez | Diffa | National |
|---|---|---|---|---|---|---|---|---|
| AMR Monitoring | $77,821.01 ($76,076.45, $81,568.04) | $86,823.57 ($84,752.59, $90,414.27) | $94,963.53 ($92,500.51, $99,510.88) | $123,018 ($120,052, $128,851) | $104,538 ($102,301, $108,884) | $35,321.95 ($34,891.61, $36,095.90) | $45,763.00 ($45,026.84, $47,294.37) | $568,249 ($555,600, $592,618) |
| Mortality Monitoring | $77,821.01 ($76,076.45, $81,568.04) | $86,823.57 ($84,752.59, $90,414.27) | $94,963.53 ($92,500.51, $99,510.88) | $123,018 ($120,052, $128,851) | $104,538 ($102,301, $108,884) | $35,321.95 ($34,891.61, $36,095.90) | $45,763.00 ($45,026.84, $47,294.37) | $568,249 ($555,600, $592,618) |
