# Supplementary material for: Micro-costing for national-scale azithromycin mass drug administration to improve child survival in Niger
Source: PLOS Glob Public Health. 2026 Jun 26;6(6):e0006039. doi: 10.1371/journal.pgph.0006039 (PMC13309011; doi:10.1371/journal.pgph.0006039)
Supplement: S6 Table — (PDF) [file pgph.0006039.s008.pdf]

Supplemental Table 6. Cross-Cutting Costs

Cost Type	Dosso	Tahoua	Maradi	Zinder	Tillaberi	Agadez	Diffa	National
Transport Costs	$286,545 ($283,212, $306,542)	$349,053 ($345,680, $369,295)	$346,091 ($341,695, $372,463)	$420,544 ($414,808, $454,958)	$395,841 ($391,596, $421,311)	$190,379 ($189,676, $194,598)	$200,296 ($198,797, $209,291)	$2,188,749 ($2,165,464, $2,328,457)
Education, information, and communication	$266,046 ($266,046, $266,046)	$244,814 ($244,814, $244,814)	$294,028 ($294,028, $294,028)	$442,531 ($442,531, $442,531)	$335,798 ($335,798, $335,798)	$80,215.94 ($80,215.94, $80,215.94)	$144,385 ($144,385, $144,385)	$1,807,819 ($1,807,819, $1,807,819)
