# Supplementary material for: Micro-costing for national-scale azithromycin mass drug administration to improve child survival in Niger
Source: PLOS Glob Public Health. 2026 Jun 26;6(6):e0006039. doi: 10.1371/journal.pgph.0006039 (PMC13309011; doi:10.1371/journal.pgph.0006039)
Supplement: Attachment — Submitted filename: Response to Reviewers.docx [file pgph.0006039.s009.docx]

**Response to Reviewers**

**“Micro-costing for national-scale azithromycin mass drug administration to improve child survival in Niger”**

**Journal Requirements:**

**1. Please provide a detailed online Financial Disclosure statement. This is published with the article. It must therefore be completed in full sentences and contain the exact wording you wish to be published.**

**a) State the initials, alongside each funding source, of each author to receive each grant. For example: “This work was supported by the National Institutes of Health (####### to AM; ###### to CJ) and the National Science Foundation (###### to AM).”**

**b) State what role the funders took in the study. If the funders had no role in your study, please state: “The funders had no role in study design, data collection and analysis, decision to publish, or preparation of the manuscript.”**

**For more information, please go to our submission guidelines:**
[**https://journals.plos.org/globalpublichealth/s/submission-guidelines#loc-financial-disclosure-statement**](https://urldefense.com/v3/__https:/journals.plos.org/globalpublichealth/s/submission-guidelines*loc-financial-disclosure-statement__;Iw!!LQC6Cpwp!vcIHfmUwuZ1kfMhBmTyMenALi-qzDQrtR34LLxG0NnSIn-H6edAqD3q1GSKb6eQlUkwM4aZcTxzasWQAhI62fKoQWoSidM76AogAG8u94Rc$)

We now include the following statement:

This work was supported by Gates Foundation (grants OPP1210548 to TL and INV-002454 to TL), the Peierls Foundation, Research to Prevent Blindness, and That Man May See. Pfizer donated azithromycin for the trial. Gates Foundation reviewed and approved the study design. The funders had no role in study implementation, data collection and analysis, decision to publish, or preparation of the manuscript.

**2. Please ensure that the funders and grant numbers match between the Financial Disclosure field and the Funding Information tab in your submission form. Note that the funders must be provided in the same order in both places as well.**

We have checked and now the information aligns in both places.

**3. Please update your online Competing Interests statement. If you have no competing interests to declare, please state: "The authors have declared that no competing interests exist."**

We have updated this.

**4. Please provide separate main figure files in .tif or .eps format only and ensure that all files are under our size limit of 10MB.**

**For more information about how to convert your figure files please see our guidelines:** [**https://journals.plos.org/globalpublichealth/s/figures**](https://urldefense.com/v3/__https:/journals.plos.org/globalpublichealth/s/figures__;!!LQC6Cpwp!vcIHfmUwuZ1kfMhBmTyMenALi-qzDQrtR34LLxG0NnSIn-H6edAqD3q1GSKb6eQlUkwM4aZcTxzasWQAhI62fKoQWoSidM76AogAUC7ZRDA$)

We have uploaded converted figures.

**5. We have noticed that you have uploaded Supporting Information files, but you have not included a list of legends. Please add a full list of legends for your Supporting Information files before or after the references list.**

We have added a list of legends.

**6. If the reviewer comments include a recommendation to cite specific previously published works, please review and evaluate these publications to determine whether they are relevant and should be cited. There is no requirement to cite these works unless the editor has indicated otherwise.** 
**7. Please review your reference list to ensure that it is complete and correct. If you have cited papers that have been retracted, please include the rationale for doing so in the manuscript text, or remove these references and replace them with relevant current references. Any changes to the reference list should be mentioned in the rebuttal letter that accompanies your revised manuscript. If you need to cite a retracted article, indicate the article’s retracted status in the References list and also include a citation and full reference for the retraction notice.**

Thank you for this information.

**Reviewer #1:**

**This is a detailed evaluation of the costs of azithromycin MDA. The methodology is sound and the paper is well written. The discussion of economies of scale is particularly strong and timely.

I have the following minor comments:
• The fact that you are focusing on financial costs should be more clearly reflected in the abstract.**

Thanks for pointing this out. We’ve explicitly added the word “financial” to the abstract, see line 48.

**• Since economic costs (rather than financial costs) are typically considered the standard in full economic evaluations, this distinction in cost types (and the implications) should be discussed more explicitly in the Discussion section, ideally as a dedicated paragraph.**

Thank you, we have added the following text to the Discussion (lines 445-452):

As noted, AZ-MDA in Niger does not rely on volunteer time, thus raising the financial cost of the program when compared to the benchmark cost. By contrast, volunteer time carries a substantial economic cost, not included within the meta-analysis benchmark due to limited reporting in the original papers. Although our study also does not capture non-financial economic costs, these are likely a small addition in our case, as few elements of the program currently involve in-kind donations (aside from the drug, whose price is reported) and the house-to-house delivery minimizes household economic burden.

**• It would be helpful to include references in the Methods for the assumed cost per dose of azithromycin.**

We agree and have added more detail regarding the source for the landed cost per dose of azithromycin (lines 295-298):

“including drug price at lower ($0.40 per dose) and higher ($0.70 per dose) benchmarks derived from regional REACH data on landed cost in order to represent replacement cost in the absence of continued drug donation (personal correspondence, D. Call)”

**• The Sensitivity Analysis section in the Results feels a little brief and could be expanded slightly.**

We agree and have added additional detail to this section, explicitly touching on each category, (lines 424-433):

To identify the types of expenditures whose cost variation might have the greatest impact on the total budget, we examined changes, both negative and positive, in per diem rates, salaries, equipment, vehicles, fuel, drug transport & storage, and communication (Figure 3). A 25% change in per diem would result in the largest change to the national budget at $846K. Adjustment to vehicle costs and fuel charges by 50% would also bring substantial changes at $349K and $242K, respectively. A 25% change to salary would impact overall cost by $233K. Similarly, a 25% change to equipment costs would bring about a $217K impact. Adjusting drug transport costs by 25% would have a $176K impact. Finally, a 25% change in communication costs would have the smallest budgetary impact at just $9K.

**• The majority of cost data were extracted from delivery trial expenditure records. Were any research costs excluded? Trials often differ from programmatic delivery, and this should be acknowledged as a potential limitation in the Discussion.**

Thanks for this suggestion. We did exclude research costs and only included those applicable for programmatic delivery. We’ve added more detail to the Methods (lines 132-136) which now state the specific costs that were excluded which were wages for the post-distribution survey team, per diem for the medical consultant, and the costs of stringent drug monitoring. We’ve also added a limitation that mentions there may be some residual trial costs included (lines 514-519):

“Another potential limitation is that while we removed all trial expenditures not applicable to a national program, there may be residual costs included here that differ from the reality of programmatic implementation. While this is a possibility, we believe our detailed monitoring of costs allowed us to parse trial specific costs from real-world implementation costs with good fidelity.”

**• It is great to see the 95% uncertainty intervals on the costs. It would be helpful to expand the Methods section to describe exactly how these were calculated. Currently, the abbreviation “UI” is only spelled out in the abstract.**

Thanks for this comment. We’ve added deeper explanation of the UI calculation in lines 314-323. Briefly, as no line-item costs were influenced by both sources simultaneously, each was calculated independently. Mobilizer proportion uncertainty via a Beta distribution with 200 simulated draws, and coverage uncertainty via the empirically-observed 95% CI from AVENIR Delivery I, with the lower and upper bounds of each applied to the relevant cost items.

**• Regarding the sentence: “Our cost per dose is higher than found in a previous meta-analysis of MDA against neglected tropical diseases, which identified a benchmark of $0.50 per dose ($0.67 per dose in 2025 dollars).” I do not believe (apologies if I’m wrong) that this study identified this benchmark — it was already commonly used. I suggest double checking the references and wording around the US$0.50 benchmark.**

Thanks for pointing this out, we’ve updated the wording to state “which pooled evidence from 56 costing studies to confirm a benchmark.”, line 444.

**• Figure 1 – please check the axis labels.**

The y axis labels the total cost in USD, which is the addition of each separate category. To make this clearer we’ve changed the x axis title to “Total Cost in USD ($)”.

**• Clearly define that you are using K for thousands and M for millions when presenting costs in the text.**

We’ve clarified this in the abstract, line 53, and in the results, lines 338-339.

**• State the method used to adjust for inflation in more detail (e.g., use of local vs. US inflation rates)**

We’ve added clarification that we inflated the CFA costs using Niger inflation rates and then converted to USD, line 138-139.

Reviewer #2: Review of Research Article PGPH-D-26-00439, Micro-costing for national-scale azithromycin mass drug administration to improve child survival in Niger
 **The manuscript describes a study to estimate the cost of implementing azithromycin mass drug administration for child survival in Niger. The study will be helpful to Niger and other countries considering this intervention. The manuscript is well written, the acquisition of cost data is thorough, and the analyses are appropriate for the research questions.**

Thank you for your compliments.

**A few comments are below.

Major:
In the first paragraph of the Results section and again in the first paragraph of the Discussion, the authors write about cost per dose. This was slightly confusing as the treatment regimen in Niger (and elsewhere) calls for double dose to children in fairly close succession. Therefore, to understand the true cost of this treatment regimen, does the dose cost need to be doubled? Or does the $1.59 represent a “treatment cost per person”? Please do clarify this in the Methods and possibly with a footnote for Table 2.**

The MDA regimen in Niger consists of single doses spaced approximately 6 months apart. We have clarified this in the methods and added a citation (line 144):

“AZ-MDA programs delivery twice-yearly treatments of a single dose per child at each time point.”

We also provide clarity on how cost per dose is calculated (line 156-158):

“Cost per dose was calculated as the estimated budget for a single year divided by the number of doses delivered across two rounds of MDA.”

**In the discussion section the authors describe M&E activities such as AMR monitoring that they expect programs to continue to implement during programmatic implementation of the MDA regimen. They also include these costs in their overall estimate. It seems that this type of monitoring (as well as the QC monitoring of drug supply, for example) may be the first to be dropped if and when programs face budget shortfalls. In the Discussion, the authors should consider pointing out the importance of these activities, and maybe demonstrate that compared to most other activities, dropping this monitoring would not provide considerable savings (if that is the case).**

Thanks for pointing this out. We agree and have added additional context in the discussion section, lines 484-488.

**The Discussion would be strengthened if the authors discussed the number one factor that affected costs per table 3 and Figure 2, the cost of purchasing the medication. Readers would benefit from learning the current status of the donation program and official commitments from Pfizer. Have end-line outcomes been developed to know when a child survival program, and thus a drug donation, may cease in these settings? If the timeline is likely long, and the cost go up considerably if there is donation, is this a serious threat to the long-term viability of this intervention?**

Thanks for this comment. Pfizer is currently continuing to donate for the national implementation in Niger. Niger Ministry of Health has defined stopping guidelines, and the AVENIR II study is testing these stopping rules. We’ve added this information to lines 528-532.

**Minor
Abstract-line 44-define mass drug administration as MDA the first time it is used.**

Updated.

**Line 107-108 should use AZ-MDA.**

Updated.

**Line 149-150 was based on the assumption from the trial- is the assumption 90% with each dose or that 90% of children do get both doses?**

Based on observed coverage in the trial, 90% of eligible children received a dose at each MDA time point. We know that these are not the exact same children, as some will age out of eligibility between MDA rounds, and many infants will be born and become eligible between rounds, as each round is spaced approximately six months apart. The cost estimates are not changed whether you assume most eligible children receive both doses or that it’s a different random sample of eligible children.

**Line 200-please list the size of the bottle of water (in ml). I wonder if it will be common for countries to purchase such large quantities of bottled water? Wouldn’t some countries likely rely on their water supply with iodine or boiling if needed?**

We’ve included the size, 500 mL, in line 204. We also included the reason water bottles are used over the country’s water supply, to ensure safe, clean drinking water is used, on lines 206-207.

**Line 201-202 are cups used for those over ages 1 year?**

Cups are used for those over 1 year and are included in the drug shipments at no additional cost, we’ve added this information in lines 208-209.

**Line 387-is there a font change in this sentence?**

Thanks for flagging this, we’ve fixed it.
