# Supplementary material for: Micro-costing for national-scale azithromycin mass drug administration to improve child survival in Niger
Source: PLOS Glob Public Health. 2026 Jun 26;6(6):e0006039. doi: 10.1371/journal.pgph.0006039 (PMC13309011; doi:10.1371/journal.pgph.0006039)
Supplement: Peer review history

# Peer review: Decision Letter 0

**DOI:** 10.1371/journal.pgph.0006039.r001

**Authors:** Abraham Flaxman

24 Mar 2026

PGPH-D-26-00439

Micro-costing for national-scale azithromycin mass drug administration to improve child survival in Niger

PLOS Global Public Health

Dear Dr. Fitzpatrick,

Thank you for submitting your manuscript to PLOS Global Public Health. After careful consideration, we feel that it has merit but does not fully meet PLOS Global Public Health’s publication criteria as it currently stands. Therefore, we invite you to submit a revised version of the manuscript that addresses the points raised during the review process.

Please submit your revised manuscript by Apr 23 2026 11:59PM. If you will need more time than this to complete your revisions, please reply to this message or contact the journal office at globalpubhealth@plos.org. When you're ready to submit your revision, log on to https://www.editorialmanager.com/pgph/ and select the 'Submissions Needing Revision' folder to locate your manuscript file.

Please include the following items when submitting your revised manuscript:

- A letter that responds to each point raised by the editor and reviewer(s). You should upload this letter as a separate file labeled 'Response to Reviewers'.
- A marked-up copy of your manuscript that highlights changes made to the original version. You should upload this as a separate file labeled 'Revised Manuscript with Track Changes'.
- An unmarked version of your revised paper without tracked changes. You should upload this as a separate file labeled 'Manuscript'.

Guidelines for resubmitting your figure files are available below the reviewer comments at the end of this letter.

We look forward to receiving your revised manuscript.

Kind regards,

Abraham D. Flaxman, Ph.D.

Academic Editor

PLOS Global Public Health

Journal Requirements:

1. Please provide a detailed online Financial Disclosure statement. This is published with the article. It must therefore be completed in full sentences and contain the exact wording you wish to be published.

a) State the initials, alongside each funding source, of each author to receive each grant. For example: “This work was supported by the National Institutes of Health (####### to AM; ###### to CJ) and the National Science Foundation (###### to AM).”

b) State what role the funders took in the study. If the funders had no role in your study, please state: “The funders had no role in study design, data collection and analysis, decision to publish, or preparation of the manuscript.”

For more information, please go to our submission guidelines:

https://journals.plos.org/globalpublichealth/s/submission-guidelines#loc-financial-disclosure-statement

2. Please ensure that the funders and grant numbers match between the Financial Disclosure field and the Funding Information tab in your submission form. Note that the funders must be provided in the same order in both places as well.

3. Please update your online Competing Interests statement. If you have no competing interests to declare, please state: "The authors have declared that no competing interests exist."

4. Please provide separate main figure files in .tif or .eps format only and ensure that all files are under our size limit of 10MB.

For more information about how to convert your figure files please see our guidelines: https://journals.plos.org/globalpublichealth/s/figures

5. We have noticed that you have uploaded Supporting Information files, but you have not included a list of legends. Please add a full list of legends for your Supporting Information files before or after the references list.

6. If the reviewer comments include a recommendation to cite specific previously published works, please review and evaluate these publications to determine whether they are relevant and should be cited. There is no requirement to cite these works unless the editor has indicated otherwise.

7. Please review your reference list to ensure that it is complete and correct. If you have cited papers that have been retracted, please include the rationale for doing so in the manuscript text, or remove these references and replace them with relevant current references. Any changes to the reference list should be mentioned in the rebuttal letter that accompanies your revised manuscript. If you need to cite a retracted article, indicate the article’s retracted status in the References list and also include a citation and full reference for the retraction notice.

Additional Editor Comments (if provided):

[Note: HTML markup is below. Please do not edit.]

Reviewers' comments:

Reviewer's Responses to Questions

**Comments to the Author**

1. Does this manuscript meet PLOS Global Public Health’s publication criteria? Is the manuscript technically sound, and do the data support the conclusions? The manuscript must describe methodologically and ethically rigorous research with conclusions that are appropriately drawn based on the data presented.

Reviewer #1: Yes

Reviewer #2: Yes

**********

2. Has the statistical analysis been performed appropriately and rigorously?

Reviewer #1: Yes

Reviewer #2: Yes

**********

3. Have the authors made all data underlying the findings in their manuscript fully available (please refer to the Data Availability Statement at the start of the manuscript PDF file)?

The PLOS Data policy requires authors to make all data underlying the findings described in their manuscript fully available without restriction, with rare exception. The data should be provided as part of the manuscript or its supporting information, or deposited to a public repository. For example, in addition to summary statistics, the data points behind means, medians and variance measures should be available. If there are restrictions on publicly sharing data—e.g. participant privacy or use of data from a third party—those must be specified.

Reviewer #1: Yes

Reviewer #2: Yes

**********

4. Is the manuscript presented in an intelligible fashion and written in standard English?

PLOS Global Public Health does not copyedit accepted manuscripts, so the language in submitted articles must be clear, correct, and unambiguous. Any typographical or grammatical errors should be corrected at revision, so please note any specific errors here.

Reviewer #1: Yes

Reviewer #2: Yes

**********

5. Review Comments to the Author

Please use the space provided to explain your answers to the questions above. You may also include additional comments for the author, including concerns about dual publication, research ethics, or publication ethics. (Please upload your review as an attachment if it exceeds 20,000 characters)

Reviewer #1: This is a detailed evaluation of the costs of azithromycin MDA. The methodology is sound and the paper is well written. The discussion of economies of scale is particularly strong and timely.

I have the following minor comments:

• The fact that you are focusing on financial costs should be more clearly reflected in the abstract.

• Since economic costs (rather than financial costs) are typically considered the standard in full economic evaluations, this distinction in cost types (and the implications) should be discussed more explicitly in the Discussion section, ideally as a dedicated paragraph.

• It would be helpful to include references in the Methods for the assumed cost per dose of azithromycin.

• The Sensitivity Analysis section in the Results feels a little brief and could be expanded slightly.

• The majority of cost data were extracted from delivery trial expenditure records. Were any research costs excluded? Trials often differ from programmatic delivery, and this should be acknowledged as a potential limitation in the Discussion.

• It is great to see the 95% uncertainty intervals on the costs. It would be helpful to expand the Methods section to describe exactly how these were calculated. Currently, the abbreviation “UI” is only spelled out in the abstract.

• Regarding the sentence: “Our cost per dose is higher than found in a previous meta-analysis of MDA against neglected tropical diseases, which identified a benchmark of $0.50 per dose ($0.67 per dose in 2025 dollars).” I do not believe (apologies if I’m wrong) that this study identified this benchmark — it was already commonly used. I suggest double checking the references and wording around the US$0.50 benchmark.

• Figure 1 – please check the axis labels.

• Clearly define that you are using K for thousands and M for millions when presenting costs in the text.

• State the method used to adjust for inflation in more detail (e.g., use of local vs. US inflation rates)

Reviewer #2: Review of Research Article PGPH-D-26-00439

Micro-costing for national-scale azithromycin mass drug administration to improve child survival in Niger

The manuscript describes a study to estimate the cost of implementing azithromycin mass drug administration for child survival in Niger. The study will be helpful to Niger and other countries considering this intervention. The manuscript is well written, the acquisition of cost data is thorough, and the analyses are appropriate for the research questions. A few comments are below.

Major:

In the first paragraph of the Results section and again in the first paragraph of the Discussion, the authors write about cost per dose. This was slightly confusing as the treatment regimen in Niger (and elsewhere) calls for double dose to children in fairly close succession. Therefore, to understand the true cost of this treatment regimen, does the dose cost need to be doubled? Or does the $1.59 represent a “treatment cost per person”? Please do clarify this in the Methods and possibly with a footnote for Table 2.

In the discussion section the authors describe M&E activities such as AMR monitoring that they expect programs to continue to implement during programmatic implementation of the MDA regimen. They also include these costs in their overall estimate. It seems that this type of monitoring (as well as the QC monitoring of drug supply, for example) may be the first to be dropped if and when programs face budget shortfalls. In the Discussion, the authors should consider pointing out the importance of these activities, and maybe demonstrate that compared to most other activities, dropping this monitoring would not provide considerable savings (if that is the case).

The Discussion would be strengthened if the authors discussed the number one factor that affected costs per table 3 and Figure 2, the cost of purchasing the medication. Readers would benefit from learning the current status of the donation program and official commitments from Pfizer. Have end-line outcomes been developed to know when a child survival program, and thus a drug donation, may cease in these settings? If the timeline is likely long, and the cost go up considerably if there is donation, is this a serious threat to the long-term viability of this intervention?

Minor

Abstract-line 44-define mass drug administration as MDA the first time it is used.

Line 107-108 should use AZ-MDA.

Line 149-150 was based on the assumption from the trial- is the assumption 90% with each dose or that 90% of children do get both doses?

Line 200-please list the size of the bottle of water (in ml). I wonder if it will be common for countries to purchase such large quantities of bottled water? Wouldn’t some countries likely rely on their water supply with iodine or boiling if needed?

Line 201-202 are cups used for those over ages 1 year?

Line 387-is there a font change in this sentence?

**********

6. PLOS authors have the option to publish the peer review history of their article (what does this mean?). If published, this will include your full peer review and any attached files.

**Do you want your identity to be public for this peer review?**  If you choose “no”, your identity will remain anonymous but your review may still be made public.

For information about this choice, including consent withdrawal, please see our Privacy Policy.

Reviewer #1: No

Reviewer #2: No

**********

[NOTE: If reviewer comments were submitted as an attachment file, they will be attached to this email and accessible via the submission site. Please log into your account, locate the manuscript record, and check for the action link "View Attachments". If this link does not appear, there are no attachment files.]

Figure Resubmissions:

While revising your submission, we strongly recommend that you use PLOS’s NAAS tool (https://ngplosjournals.pagemajik.ai/artanalysis) to test your figure files. NAAS can convert your figure files to the TIFF file type and meet basic requirements (such as print size, resolution), or provide you with a report on issues that do not meet our requirements and that NAAS cannot fix.

After uploading your figures to PLOS’s NAAS tool - https://ngplosjournals.pagemajik.ai/artanalysis, NAAS will process the files provided and display the results in the "Uploaded Files" section of the page as the processing is complete. If the uploaded figures meet our requirements (or NAAS is able to fix the files to meet our requirements), the figure will be marked as "fixed" above. If NAAS is unable to fix the files, a red "failed" label will appear above. When NAAS has confirmed that the figure files meet our requirements, please download the file via the download option, and include these NAAS processed figure files when submitting your revised manuscript.

# Author comment: Author response to Decision Letter 1

**DOI:** 10.1371/journal.pgph.0006039.r002

7 May 2026

Attachment Submitted filename: Response to Reviewers.docx

# Peer review: Decision Letter 1

**DOI:** 10.1371/journal.pgph.0006039.r003

**Authors:** Abraham Flaxman, Abraham Flaxman

31 May 2026

Micro-costing for national-scale azithromycin mass drug administration to improve child survival in Niger

PGPH-D-26-00439R1

Dear Dr Fitzpatrick,

We are pleased to inform you that your manuscript 'Micro-costing for national-scale azithromycin mass drug administration to improve child survival in Niger' has been provisionally accepted for publication in PLOS Global Public Health.

Before your manuscript can be formally accepted you will need to complete some formatting changes, which you will receive in a follow up email. A member of our team will be in touch with a set of requests.

Please note that your manuscript will not be scheduled for publication until you have made the required changes, so a swift response is appreciated.

IMPORTANT: The editorial review process is now complete. PLOS will only permit corrections to spelling, formatting or significant scientific errors from this point onwards. Requests for major changes, or any which affect the scientific understanding of your work, will cause delays to the publication date of your manuscript.

If your institution or institutions have a press office, please notify them about your upcoming paper to help maximize its impact. If they'll be preparing press materials, please inform our press team as soon as possible -- no later than 48 hours after receiving the formal acceptance. Your manuscript will remain under strict press embargo until 2 pm Eastern Time on the date of publication. For more information, please contact globalpubhealth@plos.org.

Thank you again for supporting Open Access publishing; we are looking forward to publishing your work in PLOS Global Public Health.

Best regards,

Abraham D. Flaxman, Ph.D.

Academic Editor

PLOS Global Public Health

***********************************************************

Reviewer Comments (if any, and for reference):

Reviewer's Responses to Questions

**Comments to the Author**

1. If the authors have adequately addressed your comments raised in a previous round of review and you feel that this manuscript is now acceptable for publication, you may indicate that here to bypass the “Comments to the Author” section, enter your conflict of interest statement in the “Confidential to Editor” section, and submit your "Accept" recommendation.

Reviewer #1: All comments have been addressed

Reviewer #2: All comments have been addressed

**********

2. Does this manuscript meet PLOS Global Public Health’s publication criteria? Is the manuscript technically sound, and do the data support the conclusions? The manuscript must describe methodologically and ethically rigorous research with conclusions that are appropriately drawn based on the data presented.

Reviewer #1: Yes

Reviewer #2: Yes

**********

3. Has the statistical analysis been performed appropriately and rigorously?

Reviewer #1: N/A

Reviewer #2: Yes

**********

4. Have the authors made all data underlying the findings in their manuscript fully available (please refer to the Data Availability Statement at the start of the manuscript PDF file)?

The PLOS Data policy requires authors to make all data underlying the findings described in their manuscript fully available without restriction, with rare exception. The data should be provided as part of the manuscript or its supporting information, or deposited to a public repository. For example, in addition to summary statistics, the data points behind means, medians and variance measures should be available. If there are restrictions on publicly sharing data—e.g. participant privacy or use of data from a third party—those must be specified.

Reviewer #1: Yes

Reviewer #2: Yes

**********

5. Is the manuscript presented in an intelligible fashion and written in standard English?

PLOS Global Public Health does not copyedit accepted manuscripts, so the language in submitted articles must be clear, correct, and unambiguous. Any typographical or grammatical errors should be corrected at revision, so please note any specific errors here.

Reviewer #1: Yes

Reviewer #2: Yes

**********

6. Review Comments to the Author

Please use the space provided to explain your answers to the questions above. You may also include additional comments for the author, including concerns about dual publication, research ethics, or publication ethics. (Please upload your review as an attachment if it exceeds 20,000 characters)

Reviewer #1: My comments have been addressed

Reviewer #2: (No Response)

**********

7. PLOS authors have the option to publish the peer review history of their article (what does this mean?). If published, this will include your full peer review and any attached files.

**Do you want your identity to be public for this peer review?**  If you choose “no”, your identity will remain anonymous but your review may still be made public.

For information about this choice, including consent withdrawal, please see our Privacy Policy.

Reviewer #1: No

Reviewer #2: No

**********
